# The association between self-rated health and all-cause mortality and explanatory factors in China’s oldest-old population

**DOI:** 10.7189/jogh.12.11005

**Published:** 2022-07-23

**Authors:** Shangzhi Xiong, Zhiyang Wang, Beomhyeok Lee, Qi Guo, Nicholas Peoples, Xurui Jin, Enying Gong, Yaxi Li, Xinyue Chen, Zhengting He, Xian Zhang, Lijing L Yan

**Affiliations:** 1 Global Health Research Center, Duke Kunshan University, Kunshan, Jiangsu, China; 2 The George Institute for Global Health, University of New South Wales, Sydney, New South Wales, Australia; 3 Faculty of Epidemiology and Population Health, London School of Hygiene and Tropical Medicine, London, UK; 4 Baylor College of Medicine, Houston, Texas, USA; 5 MindRank AI ltd., Hangzhou, Zheijang, China; 6 Nossal Institute for Global Health, Melbourne School of Population and Global Health, The University of Melbourne, Melbourne, Australia; 7 School of population medicine and public health, China Academy of Medical Science & Peking Union Medical College, Beijing, China; 8 Department of Epidemiology, Bloomberg School of Public Health, Johns Hopkins University, Baltimore, Maryland, USA; 9 Duke Global Health Institute, Duke University, Durham, North Carolina, USA; 10 Department of Preventive Medicine, Feinberg School of Medicine, Northwestern University, Chicago, Illinois, USA; 11 The George Institute for Global Health, Beijing, China; 12 School of Public Health, Wuhan University, Wuhan, China

## Abstract

**Background:**

Self-rated health (SRH) is considered a condensed summary of information about bodily conditions that involves people’s biological, cognitive, and cultural status, but has been under-studied in the oldest old population. This study aimed to investigate the association between SRH and all-cause mortality among the oldest-old population in China and to explore potential explanatory factors in this association.

**Methods:**

The study was based on the Chinese Longitudinal Healthy Longevity Survey (CLHLS) from 1998 to 2018 and included 30 222 participants aged 80 years or older (ie, the oldest old) in the analysis. We used Cox models to assess the association between SRH and mortality in this population and its subgroups, and used the Percentage Excess Risk Mediated approach to identify potential contributing factors.

**Results:**

After adjustment of confounders, people with “good” “neutral”, and “bad/very bad” SRH were significantly associated with 8% (95% confidence interval (CI) = 3%-13%), 23% (95% CI = 18%-29%), and 52% (95% CI = 44%-61%) higher hazard of mortality respectively, compared with those with “very good” SRH. The significant SRH-mortality associations were exclusive to men and those with at least primary education. The adjustment of “regular physical activity”, “leisure activity”, “activities of daily living (ADL)”, and “cognitive function” all led to noticeable attenuation to the SRH-mortality association, with “leisure activity” causing the most attenuation (64.9%) in the “Good SRH” group.

**Conclusions:**

Self-rated health is significantly associated with all-cause mortality among the oldest old population in China, particularly among men and the educated, and is considerably explained by regular physical activity, leisure activity, ADL, and cognitive function. We advocate the use of SRH as a simple and efficient tool in research and (potentially) health care practices.

Self-rated health (SRH), sometimes referred to as self-reported health or self-assessments of health, is considered a condensed summary of information about bodily conditions which involves biological, cognitive, and cultural aspects [1]. Numerous studies showed that SRH was a strong predictor of all-cause mortality across different cultures, races, genders, and socioeconomic statuses [2-4]. In the UK Biobank study involving 498 103 participants aged 37-73 years, SRH was found to be a stronger predictor for five-year mortality than most commonly acknowledged strong predictors for premature deaths, such as smoking, drinking, and previous disease diagnoses [5]. In low-and middle-income countries (LMICs) similar, albeit less predominant evidence has been reported. A cohort study in eight LMICs including China found a 43% increased risk of 5-year mortality for participants (≥65 years) with poor SRH compared to those with moderate SRH levels, even after full adjustments [4].

The oldest-old population (80 years or older) is an increasingly large population in China. The World Health Organization predicted that this population in China will reach over 90 million people in 2050 [6]. This population is characterized by particular health patterns due to longevity [7], including higher prevalence of multi-morbidity, prevalent and severe frailty, and higher mortality, suggesting dire needs for comprehensive and continuous health care [8-10]. The oldest-old population is largely under-represented in health research, despite facing substantial and growing health needs [11,12], with limited involvement in clinical trials and inadequate opportunities for health measurements. More reliable and efficient tools are needed to address the challenges in health management among the oldest old population.

Although SRH is regarded as a subjective indicator of health status with predictive power for mortality in the middle-to-old populations, whether its association with mortality pertains to the oldest-old population in China, and whether and how other factors were involved in this SRH-mortality association remains unknown. Successfully addressing these knowledge gaps could potentially produce useful implications in future health research and routine practices in this under-served population [13].

This study aims to bridge the mentioned research gaps by investigating the association between SRH and all-cause mortality in the oldest old population in China and to determine potential factors that may explain the associations. We further aim to determine whether these associations differ across different socioeconomic groups.

## METHODS

### Study population

We used data from the Chinese Longitudinal Healthy Longevity Survey (CLHLS), which consisted of seven waves across 1998, 2000, 2002, 2005, 2008-09, 2011-12, 2013-14, and the latest follow-up in 2018. CLHLS is a prospective, national-wide longitudinal study, with one of the largest cohorts focused on the older population. It was conducted in randomly selected cities and counties that accounted for half of all cities and counties in 23 out of 31 provinces of China, which covers over 85% of China’s population. Details of the cohort study and data quality are described elsewhere [14-16]. In this study, we considered the first wave in which the participants were enrolled as their “baseline wave”, and all the later waves they were involved in as follow-up waves. We included participants whose baseline age was 80 years or older (the oldest-old population) and excluded those who missed the SRH information.

The Research Ethics Committees of Peking University and Duke University approved the ethics of the study (IRB00001052–13074). All included participants or their legal representatives provided written consent forms in the surveys.

### Measures

In this study, SRH was measured by a single-item question asking respondents at the baseline to rate their own overall health in five categories: “very good”, “good”, “neutral”, “bad”, and “very bad”. We combined “bad” and “very bad” groups into the “bad/very bad” group due to the limited sample size in “very bad” (1.3%).

Mortality was the other primary variable of interest in the study’s survival analyses, determined by the participants’ survival status. The vital status and date of death were collected during the survey at each wave. They were based on officially issued death certificates when available, or otherwise from the next-of-kin, or local residential committees who were close to the decedents. The duration of follow-up was calculated by the time interval between the first interview date and the date of death.

Other relevant variables were identified *a priori*, including gender (male, female), ethnicity (Han, others), age, marital status (married, widow, separated/divorced/never married), occupation before the age of 60 (manual, non-manual, and never worked), education (middle school or higher, primary school, none), and residence (rural, urban) at baseline.

We further included seven “health-related factors” in the data analysis: four lifestyle-related factors (smoking, alcohol assumption, regular physical activity, diet) and leisure activity, activities of daily living (ADL), and cognitive function. ADL indicated whether participants were capable of or needed help in various daily activities. The cognitive function of participants was measured using the Chinese version of the Mini-Mental State Examination (CMMSE) [17]. These variables were all collected from participants’ baseline surveys and are described in **Table 1** (additional information in Table S1 in the **Online Supplementary Document**).

**Table 1:** Demographic characteristics of participants according to self-rated health

Characteristics	n (%)/Mean±SD					*P*-value
	**Total**	**Very good**	**Good**	**Neutral**	**Bad/very bad**	
	**n = 30 222**	**n = 3566**	**n = 12 583**	**n = 10 225**	**n = 3848**	
**Gender**						<0.001
Female	18 183 (60.2%)	1920 (53.8%)	7475 (59.4%)	6292 (61.5%)	2496 (64.9%)	
Male	12 039 (39.8%)	1646 (46.2%)	5108 (40.6%)	3933 (38.5%)	1352 (35.1%)	
**Ethnicity**						<0.001
Han	28315 (93.7%)	3405 (95.5%)	11745 (93.3%)	9513 (93.0%)	3652 (94.9%)	
Other	1907 (6.3%)	161 (4.5%)	838 (6.7%)	712 (7.0%)	196 (5.1%)	
**Residence**						<0.001
Urban	12 734 (42.1%)	1725 (48.4%)	5325 (42.3%)	4257 (41.6%)	1427 (37.1%)	
Rural	17 488 (57.9%)	1841 (51.6%)	7258 (57.7%)	5968 (58.4%)	2421 (62.9%)	
**Marriage**						<0.001
Married	5268 (17.4%)	707 (19.8%)	2192 (17.4%)	1678 (16.4%)	691 (18.0%)	
Others	24 492 (81.0%)	2828 (79.3%)	10 207 (81.1%)	8351 (81.7%)	3106 (80.7%)	
Widowed	462 (1.5%)	31 (0.9%)	184 (1.5%)	196 (1.9%)	51 (1.3%)	
**Occupation**						<0.001
Manual	28 465 (94.2%)	3246 (91.0%)	11 880 (94.4%)	9676 (94.6%)	3663 (95.2%)	
Non-manual	1752 (5.8%)	320 (9.0%)	703 (5.6%)	546 (5.3%)	183 (4.8%)	
Never worked	5 (0.0%)	0 (0.0%)	0 (0.0%)	3 (0.0%)	2 (0.1%)	
**Education**						<0.001
None	20 950 (69.3%)	2242 (62.9%)	8582 (68.2%)	7207 (70.5%)	2919 (75.9%)	
Primary School	6120 (20.3%)	806 (22.6%)	2637 (21.0%)	2047 (20.0%)	630 (16.4%)	
Middle or higher	3152 (10.4%)	518 (14.5%)	1364 (10.8%)	971 (9.5%)	299 (7.8%)	
**Smoking**						<0.001
Never smoke	21 142 (70.0%)	2415 (67.7%)	8636 (68.6%)	7332 (71.7%)	2759 (71.7%)	
Former smoker	4371 (14.5%)	513 (14.4%)	1776 (14.1%)	1455 (14.2%)	627 (16.3%)	
current smoker	4709 (15.6%)	638 (17.9%)	2171 (17.3%)	1438 (14.1%)	462 (12.0%)	
**Drinking**						<0.001
Never drank	21 257 (70.3%)	2396 (67.2%)	8675 (68.9%)	7370 (72.1%)	2816 (73.2%)	
Former drank	3156 (10.4%)	331 (9.3%)	1219 (9.7%)	1072 (10.5%)	534 (13.9%)	
Current drank lightly	2006 (6.6%)	275 (7.7%)	927 (7.4%)	656 (6.4%)	148 (3.8%)	
Current drank heavily	3803 (12.6%)	564 (15.8%)	1762 (14.0%)	1127 (11.0%)	350 (9.1%)	
**Physical activity (PA)**						<0.001
Regular PA present	7977 (26.4%)	1529 (42.9%)	3709 (29.5%)	2200 (21.5%)	539 (14.0%)	
Regular PA none	22 245 (73.6%)	2037 (57.1%)	8874 (70.5%)	8025 (78.5%)	3309 (86.0%)	
***Continuous Variable***						
**Age**	92.2 (7.4)	91.3 (7.4)	92.1 (7.4)	92.5 (7.4)	92.3 (7.5)	<0.001

SD – standard deviation

### Data analysis

We used a two-way table to summarize the demographics and socioeconomic characteristics among respondents in different SRH categories. χ2 and analysis of variance (ANOVA) tests were used to compare the categorical/continuous variables of people in different SRH categories. We used the Kaplan-Meier (KM) curve to illustrate the crude probability of survival over time by different SRH categories. Cox proportional hazard models were used to examine the associations of SRH with mortality. We tested the proportional-hazards assumption by comparing the observed survival curves to the Cox predicted curves in KM plots. The observed values were highly close to the predicted values, suggesting no violation of the assumption (Figure S1 in the **Online Supplementary Document**). There were two adjustment models: one was minimally adjusted with demographic variables, and the other was fully adjusted with both demographics and the seven health-related factors. We also conducted stratified analysis to determine whether the SRH-mortality associations differed across different gender, residence, and educational level groups (dichotomized to educated and non-educated).

To assess the extent to which each health-related factor explained the association between SRH and mortality, we used the percentage excess risk mediated (PERM), which was calculated by the following formula:

*PERM = [hazard ratio (minimally adjusted model) – hazard ration(further adjusted model] / [hazard ratio (minimally adjusted model)-1]*

The approach has been applied in previous studies to estimate the proportion reduction in the regression coefficients by health-related factors [18,19]. PERM volume for a particular group of variables could be interpreted as the proportion that the group contributed to the association between the variables of interest, regardless of the underlying mechanisms such as mediating, modifying, or confounding.

All analyses were conducted using STATA 16.1 (StataCorp, College Station, TX, USA). Regression coefficients and 95% confidence intervals (CIs) were reported.

### Ethics approval and consent to participate

The Research Ethics Committees of Peking University and Duke University approved the ethics of the study (IRB00001052–13074). All methods were carried out in accordance with The Declaration of Helsinki. All included participants or their legal representatives provided written consent forms in the surveys.

## RESULTS

### Demographic characteristic

**Figure 1:**
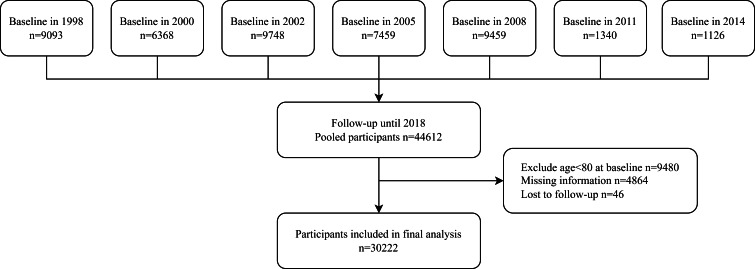
Flowchart of included participants in the study.

**Figure 1** presents the flowchart of participants included in the study from the CLHLS surveys. A total of 30 222 participants from CLHLS were included and their demographic characteristics are presented in **Table 1** (additional information in Table S1 in the **Online Supplementary Document****)**. Participants reported “very good” (11.8%), “good” (41.6%), “neutral” (33.8%), and “bad/very bad” (12.7%) SRH levels. The mean age at baseline was 92.2 years. The majority of ethnicity was Han (93.7%), and the majority of occupations were manual workers (94.2%). There were also more female participants (60.2%) than males (39.8%). The univariable analysis suggested that being a male, an urban resident, a non-manual worker, of Han ethnicity, having a higher level of education, and having regular physical activities were associated with generally better SRH (*P* < 0.001 for all). People of different smoking and drinking behaviours were significantly associated with different SRH levels, but the directions were mixed (*P* < 0.001).

### Kaplan-Meier survival curve

**Figure 2:**
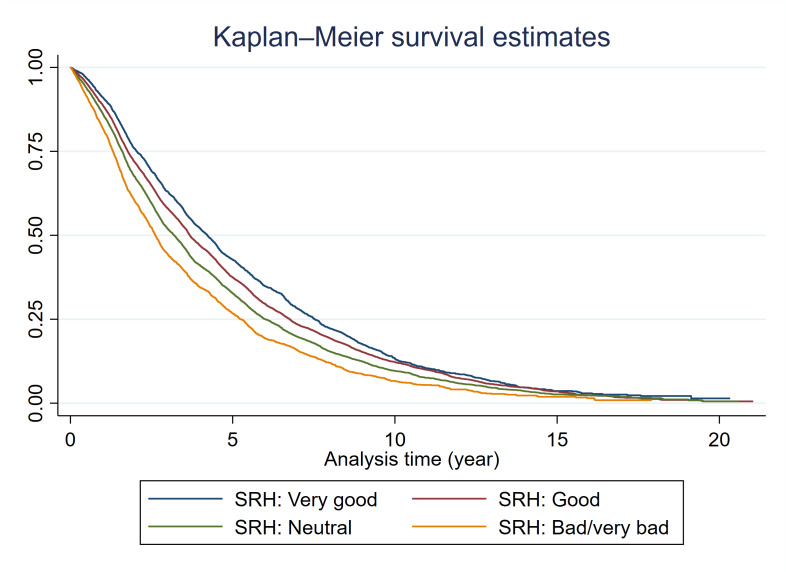
Kaplan-Meier curves by self-rated health levels.

Kaplan-Meier survival curve by SRH levels (**Figure 2**) showed that the survival curve of participants with lower SRH levels had steeper decreases than participants with better SRH. This crude association was corroborated by an observable dose-response relationship.

### Association between SRH and mortality

Results for the association between SRH and all-cause mortality were presented in **Table 2**. In the unadjusted analysis, participants with a “good/very good” (13%, 95% CI = 1.08-1.18), “neutral” (29%, 95% CI = 1.23-1.35), and “bad/very bad” (56%, 95% CI = 1.48, 1.65) SRH status had significantly higher hazard rates than those with a “very good” status. The adjustment of demographics slightly attenuated the association down to 8% (95% CI = 1.03-1.13), 23% (95% CI = 1.18-1.29), and 52% (95% CI = 1.44-1.61). However, after further adjustment of the health-related factors, only the “bad/very bad” group retained a significantly higher hazard ratio (HR) compared with the “very good” SRH group (HR = 1.13, 95% CI = 1.07-1.20).

**Table 2:** Association between self-rated health and mortality among oldest-old participants

Self-rated health	Person-year	Mortality rates	N. of death	Unadjusted HR (95% CI)	Adjusted HR (95% CI)	
				**Crude model**	Minimally adjusted*†	**Fully adjusted†**
**Main analysis**						
Very good	14 300.00	0.17	2468	Ref.	Ref.	Ref.
Good	47 038.08	0.19	9068	1.13 (1.08, 1.18)‡	1.08 (1.03, 1.13)‡	1.01 (0.96, 1.05)
Neutral	33 449.16	0.22	7255	1.29 (1.23, 1.35)‡	1.23 (1.18, 1.29)‡	1.04 (0.99, 1.09)
Bad/Very bad	11 125.49	0.26	2869	1.56 (1.48, 1.65)‡	1.52 (1.44, 1.61)‡	1.13 (1.07, 1.20)‡
**Stratified analysis**						
**In male**						
Very good	6811.36	0.16	1120	Ref.	Ref.	Ref.
Good	19 566.53	0.19	3663	1.15 (1.07, 1.23)‡	1.11 (1.04, 1.18)‡	1.03 (0.96, 1.10)
Neutral	12 959.36	0.22	2806	1.36 (1.27, 1.46)‡	1.33 (1.24, 1.42)‡	1.13 (1.05, 1.21)‡
Bad/Very bad	3663.12	0.27	990	1.75 (1.61, 1.91)‡	1.77 (1.62, 1.93)‡	1.27 (1.16, 1.40)‡
**In female**						
Very good	7488.63	0.18	1348	Ref.	Ref.	Ref.
Good	27 471.56	0.20	5405	1.10 (1.04, 1.17)‡	1.06 (0.99, 1.12)	0.99 (0.93, 1.05)
Neutral	20 539.80	0.22	4449	1.23 (1.16, 1.31)‡	1.16 (1.09, 1.24)‡	0.99 (0.93, 1.05)
Bad/Very bad	7462.37	0.25	1879	1.45 (1.35, 1.55)‡	1.39 (1.30, 1.50)‡	1.06 (0.99, 1.14)
**In urban**						
Very good	6867.68	0.16	1069	Ref.	Ref.	Ref.
Good	19 492.75	0.18	3439	1.15 (1.07, 1.23)‡	1.10 (1.03, 1.18)‡	1.01 (0.94, 1.08)
Neutral	13 459.60	0.20	2686	1.32 (1.23, 1.42)‡	1.29 (1.20, 1.38)‡	1.06 (0.99, 1.14)
Bad/Very bad	4054.69	0.24	964	1.61 (1.47, 1.75)‡	1.58 (1.45, 1.73)‡	1.10 (1.00, 1.20)‡
**In rural**						
Very good	7432.32	0.19	1399	Ref.	Ref.	Ref.
Good	27 545.34	0.20	5629	1.09 (1.03, 1.16)‡	1.06 (1.00, 1.13)‡	1.00 (0.94, 1.06)
Neutral	20 039.56	0.23	4569	1.24 (1.17, 1.31)‡	1.19 (1.12, 1.27)‡	1.03 (0.96, 1.09)
Bad/Very bad	7070.80	0.27	1905	1.49 (1.03, 1.16)‡	1.48 (1.38, 1.59)‡	1.14 (1.06, 1.23)‡
**In non-educated**						
Very good	8535.84	0.19	1640	Ref.	Ref.	Ref.
Good	31 407.54	0.20	6389	1.06 (1.01, 1.12)‡	1.03 (0.98, 1.09)	0.96 (0.91, 1.02)
Neutral	23 202.16	0.22	5207	1.19 (1.13, 1.26)‡	1.15 (1.09, 1.21)‡	0.98 (0.92, 1.04)
Bad/Very bad	8402.59	0.26	2214	1.42 (1.33, 1.51)‡	1.40 (1.31, 1.49)‡	1.06 (0.99, 1.14)
**In educated**						
Very good	5764.15	0.14	828	Ref.	Ref.	Ref.
Good	15 630.54	0.17	2679	1.21 (1.12, 1.31)‡	1.18 (1.09, 1.27)‡	1.09 (1.01, 1.18)‡
Neutral	10 297.00	0.20	2048	1.44 (1.32, 1.56)‡	1.44 (1.32, 1.56)‡	1.19 (1.10, 1.30)‡
Bad/Very bad	2722.90	0.24	655	1.78 (1.61, 1.97)‡	1.89 (1.71, 2.10)‡	1.33 (1.19, 1.48)‡

N – number, HR – hazard ratio

*Adjusted for gender, ethnicity, age, marital status, occupation, education, and residence.

†Based on minimally adjusted model, additionally adjusted for lifestyle factors (smoking, alcohol assumption, physical activity, diet, and leisure activity), ADL, and cognitive function.

‡*P* < 0.05.

In the stratified analyses (**Table 2**), we found that the significant association between SRH and mortality persisted in males but not in females after full adjustments. We also found that the association between SRH and mortality was significant only in those that were educated (had at least primary education) and not those who were non-educated. No substantial differences in the association between SRH and mortality were found between the residence subgroups. Our additional stratified analysis by the combination of gender and education groups suggested that only educated men had strongly significant associations between SRH and mortality, while no significant association was detected among non-educated men and educated or non-educated women (Table S2 in the **Online Supplementary Document**).

### Percentage excess risk mediated (PERM)

**Figure 3:**
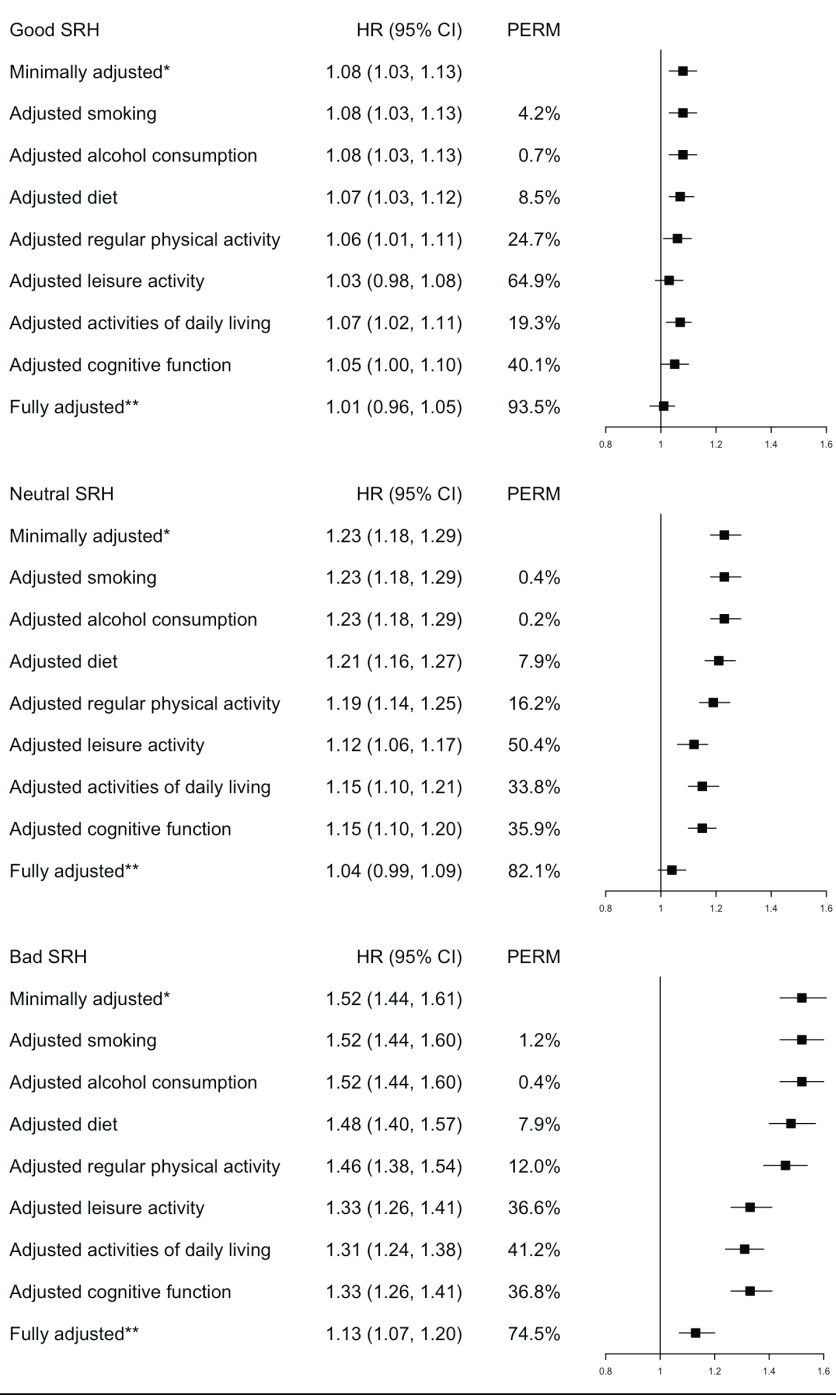
Proportions of the self-rate health (SRH)-mortality association attributable to health-related factors. Notes: The reference group was “Very good” SRH. *Adjusted for gender, ethnicity, age, marital status, occupation, education, and residence. **Adjusted for gender, ethnicity, age, marital status, occupation, education, residence, lifestyle, ADL, and cognitive function. by gender and education

As shown in **Figure 3**, the addition of any health-related factors led to a reduction in HR. The inclusion of all health-related factors in the fully adjusted model presented a substantial reduction in HR by 93.5% in the “good”, 82.1% in the “neutral”, and 74.5% in the “bad/very bad” SRH groups, with “very good” SRH being the reference.

As for individual contributions, the addition of “regular physical activity”, “leisure activity”, “ADL”, and “cognitive function” all led to noticeable reductions in HR by over 12% for the three levels of SRH, with “leisure activity” contributing to the highest reduction at 64.9% for the “good” SRH group. The addition of “alcohol consumption” contributed to the least reduction of HR by 0.7% for the “good”, 0.2% for the “neutral”, and 0.4% for the “bad/very bad” SRH levels.

## DISCUSSION

Based on CLHLS, a nationally representative cohort study of the older population in China, we found a robust association between SRH and mortality among the oldest-old population. Although the association remained significant after multiple adjustments, it was markedly attenuated by the inclusion of health-related factors (regular physical activity, leisure activity, ADL, and cognitive function). Our stratified analysis further found that after full adjustment, the significant association in this population was exclusive to men and those with at least primary education.

The significant association between SRH and mortality we found is consistent with many other studies across different cultures and populations [4,5,20]. Notably, a study in China based on data from previous waves of the same CLHLS cohort as our study also identified similar associations between SRH and mortality among those aged 65 or older, even after full adjustments [20]. Our study added to the literature that the strong association between SRH and mortality remained in China’s oldest-old population.

Our study showed that the SRH-mortality associations were influenced by people’s gender and education levels. The gender difference in the SRH-mortality associations has been documented in other studies. The UK Biobank study found SRH to be the strongest predictor for five-year mortality in men but only among the top four predictors for women [5]. However, the exact reasons behind such gender differences remained unclear. Some researchers attributed this to sex-specific reporting biases and the different disease types of men and women that underlined the documented mortality [5,21]. Notably, Benyamini et al. [21] explained that, compared with men whose SRH might be more closely linked with tangible illness, women’s SRH might be subject to a wider range of factors that are less life-threatening, which consequently neutralized its association with mortality. We further found that the significant SRH-mortality associations were exclusive to those with at least primary education. This finding was rarely documented in existing literature, but it may imply that education could empower individuals to make more accurate assessments of their own health.

A major contribution of our study is the examination of whether and the extent to which the association between SRH and mortality can be explained by health-related factors. We found that cognitive function, ADL, and lifestyle-related factors—primarily regular physical activity and leisure activities – had the most contributions to the SRH-mortality association. The important role of cognitive function in the association is plausible, since evaluating one’s own health status is by nature an active cognitive process involving considerations of their medical history, health expectation, and peer comparison [1]. Previous literature also demonstrated SRH as predictor of cognitive impairment and death [22]. ADL, on the other hand, focused more on the physical perspective, and studies showed it was significantly associated with SRH among people with chronic kidney disease [23], and with quality of life among the community-dwelling older population in South Africa and Uganda [24]. Although these existing studies did not further investigate the associations among SRH, cognitive function, ADL, and mortality, our study echoed their findings and added that the SRH-mortality association could be explained from both cognitive and physical perspectives.

The critical role of lifestyle factors in the association between SRH and mortality has been inadequately addressed in existing literature. Some studies, nevertheless, explored the associations between SRH and lifestyle per se and produced mixed findings. A cross-sectional study among older Japanese adults found various types of leisure activities to be associated with SRH across gender and work status [25]. In a Danish population aged 30-60, Pisinger et al. [26] found positive associations between physical activity, healthy diet, and SRH. The relationship between alcohol consumption and SRH, however, was U-shaped. They also did not find a significant association between people’s quitting smoking with better SRH, which they explained with the “sick quitters” effect – those who perceived themselves as “sicker” had a higher probability of smoking cessation [26]. These existing studies concurred with our PERM analysis, where we found leisure activity and regular physical activity contributed markedly to the SRH-mortality association, while smoking and drinking did not. However, these mixed findings with potentially reversed causality masked the underlying causal pathways across SRH, lifestyle factors, and mortality.

Besides SRH’s relationships with lifestyle factors, a study in Europe found that SRH was also significantly associated with people’s mental health and social functioning [27]. In another study among adults aged 20-50 in the United States, researchers found that SRH was significantly associated with not only people’s biomarkers and lifestyles, but also socioeconomic factors such as race/ethnicity and income, and environmental factors such as access to health care [13].

Although existing evidence does not clearly delineate the causal pathways among SRH, mortality, and other related factors, studies do consistently imply that SRH is an informative and “inclusive” indicator that not only reflects people’s current health status, but also their exposure to relevant coping resources and health-related behaviours, which could ultimately affect health outcomes [28]. Based on our findings, we call for more empirical studies to conduct further investigations on the underlying causal pathways among these factors.

The comparative advantages of SRH to other health indicators in health care practices are acknowledged. Benyamini et al. [28] summarized the strengths of using SRH for health evaluations as being easy to administer, cost-saving for time and money, and free of participant fatigue due to simplicity. A counterargument, however, is that SRH as a single-item assessment might be subject to suboptimal internal reliability and the advantages may not outweigh the loss of information when compared with more comprehensive health examinations [28]. Nevertheless, given the robustness of evidence for SRH’s potential predictive value for mortality and its associations with people’s behaviors, especially in the under-represented older population, the cost of adding an SRH question in a research or clinical contact is marginal in comparison to the value of the information captured.

Optimal ways to utilize the information of SRH in real-world health practices, however, need more concrete explorations. Feng et al. [20] suggested the combined use of people’s SRH and interviewer-rated health to produce a more accurate health assessment at a relatively low cost. Gallagher et al. [13] suggested the use of SRH for community-level screening to identify vulnerable individuals and neighbourhoods to address health disparities. In the context of China’s health system, with the ongoing task-shifting of population health management from hospitals to community-dwelling primary health care providers [29], further validation and utilization of SRH as a convenient tool and reasonable proxy for people’s health status could potentially generate meaningful insights for public health management, such as high-risk population identification and personalized health behaviour modification. This could be particularly meaningful for the oldest old population, given their current under-representativeness and challenges in health care.

For strengths of this study, the large, longitudinal, and nationally representative sample increased its scientific rigour. For limitations, many of the included variables of the study were self-reported (eg, the lifestyle-related factors). We tried to mitigate this by ensuring the questions’ clarity and comprehensibility for the respondents during survey dissemination. Another study limitation is that some participants in the CLHLS surveys were not included in our analysis due to the missing information on SRH. To address this, our additional descriptive analysis showed that the excluded participants shared similar sociodemographic patterns as the included participants, suggesting no detectable selection bias (Table S2 in the **Online Supplementary Document**).

## CONCLUSIONS

SRH is strongly associated with mortality among the oldest-old population in China, particularly among men and those with at least primary education. Regular physical activity, leisure activity, ADL, and cognitive function contributed largely to the association. We advocate the use of SRH as a simple and cost-effective tool in health research and potentially routine health practices. Further endeavors are needed to explore the underlying mechanisms of the relationship between SRH and health outcomes.

## Additional material


Online Supplementary Document
