# Supplementary material for: The association between self-rated health and all-cause mortality and explanatory factors in China’s oldest-old population
Source: J Glob Health. 2022 Jul 23;12:11005. doi: 10.7189/jogh.12.11005 (PMC9305379; doi:10.7189/jogh.12.11005)

Table S1: Characteristics of health-related factors of included participants by self-rated health category

Characteristics	N (%) / Mean ± SD				
	Total	Very Good	Good	Neutral	Bad/very bad
	n=30222	n=3566	n=12583	n=10225	n=3848
**Diet**	10.8 (2.7)	11.6 (2.7)	11.1 (2.7)	10.6 (2.7)	9.9 (2.8)
Fruit (n=30217)					
Rarely or never	7947 (26.3%)	652 (18.3%)	2856 (22.7%)	2876 (28.1%)	1563 (40.6%)
Occasionally	13750 (45.5%)	1479 (41.5%)	5843 (46.4%)	4885 (47.8%)	1543 (40.1%)
Everyday or except winter	8520 (28.2%)	1432 (40.2%)	3883 (30.9%)	2464 (24.1%)	741 (19.3%)
Vegetables (n=30213)					
Rarely or never	1132 (3.7%)	83 (2.3%)	330 (2.6%)	434 (4.2%)	285 (7.4%)
Occasionally	4238 (14.0%)	384 (10.8%)	1496 (11.9%)	1673 (16.4%)	685 (17.8%)
Everyday or except winter	24843 (82.2%)	3097 (86.9%)	10753 (85.5%)	8115 (79.4%)	2878 (74.8%)
Garlic (n= 30103)					
Rarely or never	12475 (41.4%)	1378 (38.8%)	5014 (40.0%)	4352 (42.7%)	1731 (45.2%)
Occasionally	13249 (44.0%)	1504 (42.4%)	5664 (45.2%)	4517 (44.3%)	1564 (40.8%)
Everyday or except winter	4379 (14.5%)	666 (18.8%)	1861 (14.8%)	1317 (12.9%)	535 (14.0%)
Meat (n= 30203)					
Rarely or never	5260 (17.4%)	612 (17.2%)	1902 (15.1%)	1768 (17.3%)	978 (25.4%)
Occasionally	15249 (50.5%)	1617 (45.4%)	6252 (49.7%)	5488 (53.7%)	1892 (49.2%)
Everyday or except winter	9694 (32.1%)	1334 (37.4%)	4418 (35.1%)	2966 (29.0%)	976 (25.4%)
Fish (n=30186)					
Rarely or never	8737 (28.9%)	858 (24.1%)	3406 (27.1%)	2940 (28.8%)	1533 (39.9%)
Occasionally	17121 (56.7%)	1990 (55.9%)	7220 (57.5%)	6000 (58.7%)	1911 (49.7%)
Everyday or except winter	4328 (14.3%)	712 (20.0%)	1936 (15.4%)	1280 (12.5%)	400 (10.4%)
Egg (n=30207)					
Rarely or never	4460 (14.8%)	434 (12.2%)	1664 (13.2%)	1566 (15.3%)	796 (20.7%)
Occasionally	14760 (48.9%)	1516 (42.5%)	5955 (47.3%)	5372 (52.6%)	1917 (49.8%)
Everyday or except winter	10987 (36.4%)	1614 (45.3%)	4960 (39.4%)	3280 (32.1%)	1133 (29.5%)
Bean (n=30216)					
Rarely or never	4675 (15.5%)	450 (12.6%)	1773 (14.1%)	1610 (15.7%)	842 (21.9%)
Occasionally	17549 (58.1%)	1813 (50.8%)	7366 (58.6%)	6151 (60.2%)	2219 (57.7%)
Everyday or except winter	7992 (26.4%)	1303 (36.5%)	3440 (27.3%)	2464 (24.1%)	785 (20.4%)
Tea (n=29952)					
Rarely or never	16666 (55.6%)	1799 (50.9%)	6810 (54.6%)	5700 (56.2%)	2357 (61.9%)
Occasionally	5426 (18.1%)	619 (17.5%)	2267 (18.2%)	1930 (19.0%)	610 (16.0%)
Everyday or except winter	7860 (26.2%)	1115 (31.6%)	3401 (27.3%)	2504 (24.7%)	840 (22.1%)
Sugar (n=30189)					
Rarely or never	7550 (25.0%)	1093 (30.7%)	3373 (26.8%)	2252 (22.0%)	832 (21.6%)
Occasionally	13194 (43.7%)	1399 (39.3%)	5549 (44.1%)	4613 (45.2%)	1633 (42.5%)
Everyday or except winter	9445 (31.3%)	1066 (30.0%)	3647 (29.0%)	3352 (32.8%)	1380 (35.9%)
Salt vegetable (n=30171)					
Rarely or never	6403 (21.2%)	817 (23.0%)	2641 (21.0%)	2093 (20.5%)	852 (22.2%)
Occasionally	10577 (35.1%)	1227 (34.5%)	4461 (35.5%)	3666 (35.9%)	1223 (31.8%)

Everyday or except winter	13191 (43.7%)	1515 (42.6%)	5460 (43.5%)	4447 (43.6%)	1769 (46.0%)
**Leisure activity**	10.2 (2.6)	11.3 (2.8)	10.5 (2.6)	9.9 (2.5)	9.3 (2.4)
Housework (n=30221)					
Never	16716 (55.3%)	1706 (47.8%)	6479 (51.5%)	5961 (58.3%)	2570 (66.8%)
Sometimes	5051 (16.7%)	633 (17.8%)	2301 (18.3%)	1724 (16.9%)	393 (10.2%)
Almost everyday	8454 (28.0%)	1227 (34.4%)	3803 (30.2%)	2539 (24.8%)	885 (23.0%)
Fieldwork (n=30219)					
Never	15847 (52.4%)	1500 (42.1%)	6203 (49.3%)	5671 (55.5%)	2473 (64.3%)
Sometimes	5872 (19.4%)	640 (18.0%)	2444 (19.4%)	2138 (20.9%)	650 (16.9%)
Almost everyday	8500 (28.1%)	1425 (40.0%)	3936 (31.3%)	2414 (23.6%)	725 (18.8%)
Garden work (n=30220)					
Never	27136 (89.8%)	2932 (82.2%)	11252 (89.4%)	9327 (91.2%)	3625 (94.2%)
Sometimes	1479 (4.9%)	270 (7.6%)	645 (5.1%)	462 (4.5%)	102 (2.7%)
Almost everyday	1605 (5.3%)	363 (10.2%)	686 (5.5%)	435 (4.3%)	121 (3.1%)
Reading (n= 30221)					
Never	25725 (85.1%)	2767 (77.6%)	10545 (83.8%)	8873 (86.8%)	3540 (92.0%)
Sometimes	2015 (6.7%)	298 (8.4%)	931 (7.4%)	648 (6.3%)	138 (3.6%)
Almost everyday	2481 (8.2%)	501 (14.0%)	1106 (8.8%)	704 (6.9%)	170 (4.4%)
Pets (n=30218)					
Never	24261 (80.3%)	2731 (76.6%)	9925 (78.9%)	8330 (81.5%)	3275 (85.1%)
Sometimes	2818 (9.3%)	366 (10.3%)	1213 (9.6%)	971 (9.5%)	268 (7.0%)
Almost everyday	3139 (10.4%)	468 (13.1%)	1444 (11.5%)	922 (9.0%)	305 (7.9%)
Mahjong (n=30220)					
Never	26427 (87.4%)	2906 (81.5%)	10744 (85.4%)	9163 (89.6%)	3614 (93.9%)
Sometimes	2626 (8.7%)	402 (11.3%)	1267 (10.1%)	784 (7.7%)	173 (4.5%)
Almost everyday	1167 (3.9%)	258 (7.2%)	572 (4.5%)	276 (2.7%)	61 (1.6%)
TV/Radio (n=30221)					
Never	12662 (41.9%)	992 (27.8%)	4806 (38.2%)	4659 (45.6%)	2205 (57.3%)
Sometimes	7823 (25.9%)	892 (25.0%)	3433 (27.3%)	2693 (26.3%)	805 (20.9%)
Almost everyday	9736 (32.2%)	1682 (47.2%)	4344 (34.5%)	2872 (28.1%)	838 (21.8%)
**Activity of daily living**	0.8 (1.5)	0.4 (0.9)	0.5 (1.2)	0.9 (1.6)	1.7 (2.1)
Bathing (n=30180)					
Unimpaired	21875 (72.5%)	2868 (80.6%)	9749 (77.6%)	7081 (69.3%)	2177 (56.7%)
Impaired	8305 (27.5%)	692 (19.4%)	2822 (22.4%)	3130 (30.7%)	1661 (43.3%)
Dressing (n=30217)					
Unimpaired	26333 (87.1%)	3397 (95.3%)	11574 (92.0%)	8632 (84.4%)	2730 (71.0%)
Impaired	3884 (12.9%)	168 (4.7%)	1009 (8.0%)	1590 (15.6%)	1117 (29.0%)
Toileting (n=30220)					
Unimpaired	25993 (86.0%)	3389 (95.0%)	11471 (91.2%)	8494 (83.1%)	2639 (68.6%)
Impaired	4227 (14.0%)	177 (5.0%)	1112 (8.8%)	1729 (16.9%)	1209 (31.4%)
Indoor transferring (n=30195)					
Unimpaired	26805 (88.8%)	3435 (96.4%)	11727 (93.3%)	8845 (86.6%)	2798 (72.8%)
Impaired	3390 (11.2%)	128 (3.6%)	841 (6.7%)	1373 (13.4%)	1048 (27.2%)
Continence (n=30219)					
Unimpaired	28314 (93.7%)	3475 (97.4%)	12104 (96.2%)	9460 (92.5%)	3275 (85.1%)
Impaired	1905 (6.3%)	91 (2.6%)	477 (3.8%)	765 (7.5%)	572 (14.9%)

Feeding (n=30214)					
Unimpaired	27702 (91.7%)	3469 (97.3%)	11971 (95.2%)	9198 (90.0%)	3064 (79.7%)
Impaired	2512 (8.3%)	97 (2.7%)	608 (4.8%)	1025 (10.0%)	782 (20.3%)
**Cognitive function score** [b]	22.0 (7.6)	24.8 (6.1)	23.0 (7.0)	21.2 (7.7)	18.3 (8.5)

a: We allowed maximum two missing values for each participant in die, leisure activities and activity of daily living.

b: The cognitive function consists of 24 items within 6 dimensions: 5 items for Orientation, 3 for Registration, 1 for Naming, 5 for Attention and Calculation, 3 for Recall and 7 for Language. The total score ranges from 0 to 30 points

Table S2: Demographic characteristics of the included and excluded participants

Characteristics	N (%) / Mean ± SD	
	Participants Included (n=30222)	Participants Excluded (n=4846)
Categorical Variable		
**Gender**		
Female	18183 (60.2%)	3536 (73.0%)
Male	12039 (39.8%)	1310 (27.0%)
**Ethnicity**		
Han	28315 (93.7%)	4539 (95.5%)
Other	1907 (6.3%)	213 (4.5%)
**Residence (no m)**		
Urban	12734 (42.1%)	1894 (39.1%)
Rural	17488 (57.9%)	2952 (60.9%)
**Marriage**		
Married	5268 (17.4%)	399 (8.3%)
Others	24492 (81.0%)	4230 (87.7%)
Widowed	462 (1.5%)	196 (4.1%)
**Occupation**		
Manual	28465 (94.2%)	4565 (97.0%)
Non-manual	1752 (5.8%)	141 (3.0%)
Never worked	5 (0.0%)	0
**Education**		
None	20950 (69.3%)	3750 (82.3%)
Primary School	6120 (20.3%)	554 (12.2%)
Middle or higher	3152 (10.4%)	255 (5.6%)
**Self-Rated Health**		
Very Good	3566 (11.8%)	72 (9.1%)
Good	12583 (41.6%)	325 (41.0%)
Neutral	10225 (33.8%)	295 (37.2%)
Bad	3848 (12.7%)	101 (12.7%)
Continuous Variable		
**Age**	92.2 (7.4)	97.1 (6.4)

Table S3: Stratification analysis between self-rated health and mortality by gender and education

Self-rated health	Person-year	Mortality rates	N. of death	Unadjusted HR (95%CI)	Adjusted HR (95%CI)	
				Crude model	Minimally adjusted [a]	Fully adjusted [b]
**In non-educated & male (n=4990)**						
Very good	2224.82	0.20	454	Ref.	Ref.	Ref.
Good	7437.93	0.21	1532	1.01 (0.91, 1.12)	1.00 (0.90, 1.10)	0.93 (0.84, 1.03)
Neutral	5420.58	0.23	1228	1.13 (1.01, 1.26)*	1.12 (1.00, 1.24)*	0.97 (0.87, 1.09)
Bad/Very bad	1765.13	0.29	512	1.49 (1.31, 1.69)*	1.50 (1.32, 1.70)*	1.12 (0.98, 1.28)
**In educated & male (n=7049)**						
Very good	4586.54	0.15	666	Ref.	Ref.	Ref.
Good	12128.60	0.18	2131	1.23 (1.13, 1.34)*	1.19 (1.09, 1.30)*	1.09 (1.00, 1.19)
Neutral	7538.78	0.21	1578	1.50 (1.37, 1.65)*	1.51 (1.38, 1.66)*	1.26 (1.15, 1.39)*
Bad/Very bad	1897.98	0.25	478	1.87 (1.66, 2.10)*	2.02 (1.80, 2.28)*	1.43 (1.26, 1.62)*
**In non-educated & female (n=15960)**						
Very good	6311.02	0.19	1186	Ref.	Ref.	Ref.
Good	23969.62	0.20	4857	1.08 (1.02, 1.16)*	1.04 (0.98, 1.11)	0.98 (0.92, 1.04)
Neutral	17781.58	0.22	3979	1.22 (1.14, 1.30)*	1.16 (1.09, 1.24)*	0.99 (0.92, 1.05)
Bad/Very bad	6637.45	0.26	1702	1.41 (1.31, 1.52)*	1.38 (1.28, 1.48)*	1.06 (0.98, 1.14)
**In educated & female (n=2223)**						
Very good	1177.61	0.14	162	Ref.	Ref.	Ref.
Good	3501.94	0.16	548	1.16 (0.97, 1.38)	1.16 (0.97, 1.38)	1.08 (0.91, 1.29)
Neutral	2758.22	0.17	470	1.27 (1.06, 1.52)*	1.18 (0.98, 1.41)	0.97 (0.81, 1.17)
Bad/Very bad	824.92	0.21	177	1.61 (1.30, 2.00)*	1.53 (1.24, 1.90)*	1.09 (0.87, 1.37)

a: Adjusted for gender, ethnicity, age, marital status, occupation, education, and residence.

b: Adjusted for gender, ethnicity, age, marital status, occupation, education, residence, lifestyle (smoking, alcohol assumption, physical activity, diet, and leisure activity), ADL, and cognitive function。

*: P-value < 0.05

Figure S1: Kaplan–Meier observed survival curves and Cox predicted curves

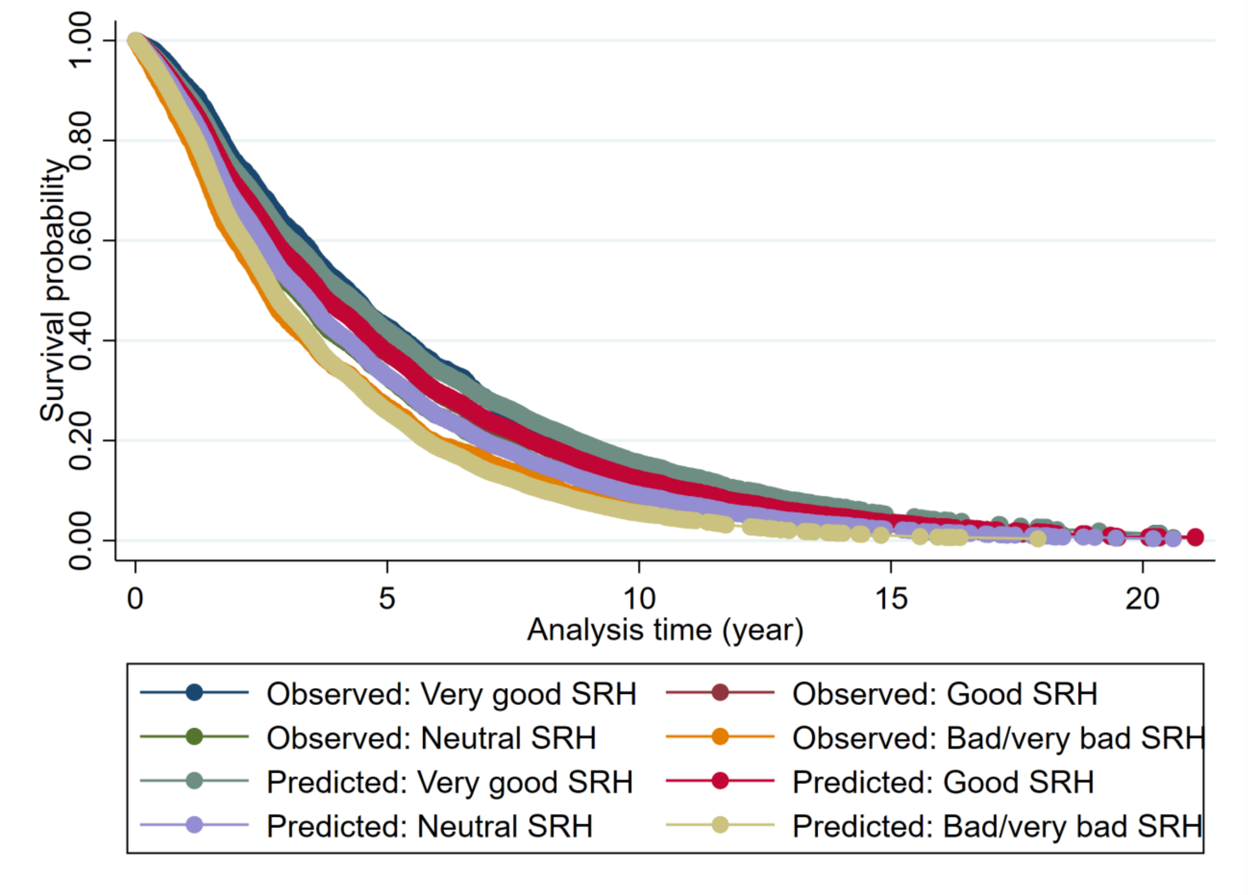

Supplement: Online Supplementary Document [file jogh-12-11005-s001.pdf]
